# Liking Food Less: The Impact of Social Influence on Food Liking Evaluations in Female Students

**DOI:** 10.1371/journal.pone.0048858

**Published:** 2012-11-14

**Authors:** Eric Robinson, Suzanne Higgs

**Affiliations:** School of Psychology, University of Birmingham, Birmingham, United Kingdom; University of Tokyo, Japan

## Abstract

Social factors are known to influence food intake and choice. However, whether social influence acts on evaluations of food and drink liking has not been studied. Across two studies, we tested whether leading a participant to believe that other people do not like a food affects food liking evaluations. In Study 1, we exposed participants to social normative information suggesting a) that an in-group disliked orange juice, b) that an out-group disliked orange juice or c) that an in-group were neutral about orange juice. We then examined how much participants believed they liked orange juice. In Study 2, participants consumed a snack food before being led to believe that two previous participants had also eaten the food and either disliked or quite liked it. We asked participants to rate how much they had enjoyed eating the snack food. Across both studies, social influence was observed, as underlined by decreases in liking evaluations. In Study 1, beliefs about liking were only influenced by social normative information when the norm was expressed by an in-group. In Study 2, exposure to others' accounts of a negative experience with a food decreased evaluated liking of the recent consumption experience. These results suggest that social influence can act upon food liking evaluations.

## Introduction

The foods we choose to eat have a significant impact on health and well-being [1] and so it is important to understand the factors that influence food choice. Liking is a good predictor of food choice and this relationship may be mediated by evaluations of which foods we think will be enjoyable to consume [2], [3]. In line with this notion, we have shown that leading participants to believe they enjoyed a food more than they actually did increased future choice and intake of that food [4]. Food liking evaluations are influenced by experienced pleasure and conditioning [5], [6] but cognitions are also important [7], [8]. For example, exposure to a food advertisement can alter evaluated enjoyment of eating the advertised food [7]. Another likely cognitive factor influencing food liking evaluations are socially transmitted stereotypes about foods [9], [10]. The popular belief that healthy foods are less enjoyable than unhealthy foods may serve as an example of this. For instance, in a study by Raghunathan [9], participants who had reported enjoying eating a food said they liked the food less when they were subsequently informed that it was healthy, which is likely to have been caused by the widely held stereotype that healthy foods lack palatability [9].

The possible influence that other people can have on evaluations about food liking has yet to be examined. Nevertheless, research indicates that both food choice and intake can be influenced by the behaviour of others present during an eating occasion [11], [12]. Social learning theories support the notion that when experiencing novel foods, young children use the willingness of others to consume the food as an indication of whether they too should eat the novel food [13], [14]. In adults, the facial reactions of dining partners have also been reported to influence how much we desire to eat the food they are eating [15]. Moreover, expectations about how enjoyable a food will be are elevated if it is believed that others have enjoyed eating that food. [16]. As well as the influence that a present eating companion can have on eating, other research also supports the notion that social normative information (the assumed dominant pattern of behaviour or preference in a social group) can alter food intake [17], [18] and inform habitual food choice [19].

Studies to date confirm that social influence can act upon some eating behaviours. Here we ask whether social influence acts on food liking evaluations. In particular, if we perceive that others dislike a food, does this affect beliefs about how much we say we like that food and is this true even for foods that we are already familiar with and have consumed recently? The social psychological literature indicates that in some contexts we look outwards and use the attitudes and appraisals of similar others to inform our own judgements [20], [21]. However, it has been suggested that such effects are most likely in contexts in which we lack knowledge or there is uncertainty [22], [9]. Moreover, there is some thought that although people will look to others for guidance on judgements that are matters of fact, judgements concerning preferences may be less prone to social influence [23], [24], [18]. This line of reasoning suggests that as preference and liking evaluations are a matter of personal opinion (and therefore they cannot be correct or incorrect), food liking evaluations may be resistant to social influence [18].

These considerations aside, recent research suggests that food liking evaluations may be prone to social influence. Studies of laboratory animals have shown that food preferences in rats are altered after viewing another rat consuming a food, even when the food in question has been sampled previously and a stable preference has been formed [25], [26]. Moreover, studies of social norms in young adult humans have shown that perceived social norms are predictive of habitual food choice (an indirect measurement of preference) [19]. The implication from these studies is that social influence on eating behaviours may be evident even when a food has acquired a preference and has been sampled recently.

In the present studies, we explored whether social influence acts upon food liking evaluations. In Study 1, we examined the effect of social normative information about orange juice (suggesting that others dislike it) on participants' beliefs about how much they liked orange juice. In Study 1, we also examined whether social group status (in- vs. out-group status) affected social influence. We exposed participants to negative social normative information about a food. This information came from either an in-group that participants would identify with (fellow female university students) or an out-group that participants would not identify with (fellow overweight and obese male university students). In line with existing literature suggesting that association to the reference group predicts conformity [27], [28], we hypothesised that social influence would only occur in the in-group condition. The inclusion of an out-group condition allowed us to determine whether any observed effects could be explained through exposure to negative information about orange juice.

In Study 2, we examined the effect of fictitious reports of previous participants' negative experience with a food on liking evaluations. The target food had been consumed recently by the real participants. Food choices are likely to be influenced by both general beliefs about food liking and beliefs about liking for specific eating experiences [29], [30]. Thus, we wanted to explore if there would be evidence for social influence on both types of liking evaluations.

We hypothesised that across both studies, social information would affect liking evaluations. In Study 1, we predicted that participants would alter their liking evaluations in line with the negative social normative information about the in-group. In Study 2, we predicted that participants would alter their liking evaluations of a recently consumed food to be in line with the accounts of others' negative experiences with that food.

## Study 1: Method

### Overview

We led participants (female undergraduate psychology students) to believe that they would be participating in a study examining the influence of personality on perceptions of what other people do and do not like. Participants were first shown an example of some fictional results we had obtained from a previous survey. This was a cover story to expose participants to information about how much other students like orange juice. Participants read that other female students dislike orange juice (*negative in-group condition*), quite like orange juice (*neutral in-group condition*) or that a group of dissimilar students, overweight male students, dislike orange juice (*negative out-group condition*). Participants then completed a personality measure and filler questionnaire and were told that the study had been completed. As the participant was leaving, the researcher asked them if they would mind filling out a short questionnaire for use in a different study. This questionnaire included a measurement of orange juice liking. We hypothesised that liking for orange juice would be reduced after participants were exposed to information suggesting that others dislike orange juice, but only if the information came from an in-group.

### Participants

Eighty-nine female psychology undergraduate students participated in exchange for course credit (M age = 19.2 years, SD = 0.9 years). To disguise the nature of the research, the study was advertised as research examining the question ‘Does personality influence perception of others' preferences?’ The University of Birmingham Research Ethics Committee approved the study and we obtained the written consent of all participants. The study was conducted according to the ethical standards laid down in the Declaration of Helsinki 1964.

### Experimental Conditions

All participants read mock opinions about orange juice from University of Birmingham students; they were led to believe that this information was taken from a recent survey we had conducted with 100 students, in which we asked the students to taste and rate orange juice and report how often they drank it. The content of information differed by condition.

#### Negative in-group condition

Participants in this condition read that the mean self-reported liking score of orange juice = 3.2/10. Median liking score = 3/10. Percentage reporting regular consumption = 16%. Percentage rating consumed orange juice as very enjoyable = 9%. Two written evaluations: ‘I can put up with the taste’ and ‘Don't drink it, not that nice’.

#### Neutral in-group condition

Participants in this condition read that the mean self-reported liking score of orange juice = 6.2/10. Median liking score = 6/10. Percentage reporting regular consumption = 50%. Percentage rating consumed orange juice as very enjoyable = 50%. Two written evaluations: ‘The taste is OK’ and ‘It is fairly refreshing’.

#### Negative out-group condition

Participants in this condition received the exact same information as the *negative in-group condition*, but they were told that the survey had been conducted on 100 overweight and obese male students from the University of Birmingham.

### Procedure

Participants were greeted by an experimenter and taken to a testing room alone. After signing for consent, participants were given a questionnaire explaining the study. They were told that the study was about the links between personality and knowing what other people like and dislike and that they would be required to use line scales to complete answers. They were then given an *‘example’* question to practice using a line scale and were instructed to mark their answer along the line with an X; ‘How much do you think other students like orange juice?’ (10 cm line scale, anchors ‘don't like at all’ to ‘like very much’). In the negative dissimilar condition this question read ‘how much do you think overweight male students like orange juice?’ This question served as a measure of baseline beliefs about other students' liking of orange juice. Participants were then told that they would be required to rate several questions similar to the example and that in a later study the research team would ask other students about how much they like the various foods. Participants were then shown an example of some information from a previous study (the information differed by condition: see ‘Experimental Conditions’).

Participants were told that the study would require them to complete short personality measures before estimating other students’ likes and dislikes. Before doing this, the participants were asked to write down the information they had just read and complete the *example* question again to confirm they had read the information and instructions carefully; ‘How much do you think other students like orange juice?’ (10 cm line scale, anchors ‘Don't like at all’ to ‘Like very much’). This was completed as a measure of whether perceptions of peers' liking of orange juice changed as a result of reading the manipulation information. Participants next reported their age, sex and rated their hunger (‘how hungry are you right now?’ 10 cm line scale, anchors ‘not at all’ to ‘extremely’). Participants then completed 8 five-point likert scale mock personality measures (e.g. ‘I enjoy a challenge in life’). As part of the cover story, participants were asked to rate (using 10 cm line scales, anchors: ‘not at all enjoyable’ and ‘extremely enjoyable’) how much they thought other students enjoyed eating 6 foods; chocolate, pizza, popcorn, alcohol, cookies and vegetables. Participants then self-reported weight (kg) and height (metres) and were then told that the study was finished.

The researcher thanked participants for their time and as they left the room the researcher asked if they would mind completing a short questionnaire for data being collected as part of a different study (all participants agreed). Participants were asked ‘how much do you like…’ (using 10 cm line scale, anchors: ‘don't like at all’ to ‘like very much’) for 6 food and drink items; cheese, orange juice, curry, junk food, french fries and apple juice. Participant liking ratings for orange juice was the main variable of interest, although we also included apple juice to test whether any between-group differences for liking were specific for organge juice. The additional food items were included to disguise our interest in orange juice. To assess whether any participants linked the two studies, the researcher asked participants to write down the aims of the research. Participants were then fully debriefed and thanked for their participation.

### Analysis

We conducted a one-way ANOVA to examine whether the conditions differed on baseline beliefs about other students' liking of orange juice. As a check of whether the manipulation affected beliefs about other students' liking of orange juice, we computed a measure of change in belief about other students' liking of orange juice by subtracting a participant's second response concerning other students' orange liking from their first response. Thus, if participants initially rated other students as liking orange juice 7/10 but as a result of the manipulation then rated it as 3/10, they would be assigned a change in belief of other students' liking score of -4. We subjected this measure to ANOVA to compare conditions. We also conducted ANOVA to examine between-group differences for evaluations about orange juice liking and apple juice liking. Based on the existing social influence and conformity literature, we had an a-priori hypothesis that self-beliefs about orange juice liking would be decreased in the negative in- group condition vs. negative out-group and neutral in-group conditions. Based on these considerations we planned pairwise comparisons to follow up a main effect.

## Study 1: Results

Five participants came close to guessing the aims of the study and so were removed from analyses (the pattern of results did not change if they were included). The mean baseline hunger (10 cm line scale) was 5.1 (SD = 2.3) and BMI (based on self-report data) was 21.4 (SD = 3.2).

### Beliefs about others liking of orange juice

ANOVA indicated a significant effect of condition on baseline beliefs about other students' liking of orange juice [F (2,81) = 10.7, p<0.05]. There was no significant difference between the neutral and negative in-group conditions [p = 0.52], but the negative out-group condition had a significantly lower rating than the neutral [p<0.05] and negative in-group conditions [p<0.05], suggesting that at baseline, participants believed that overweight male students liked orange juice less than the two other groups. See Table 1.

**Table 1:** Study 1 Liking Ratings.

	Neutral in-group condition, n = 28	Negative in-group condition, n = 27	Negative out-group condition, n = 29
**Baseline beliefs about other students**' **orange juice liking**	7.0 (0.9)	6.8 (1.3)	5.4 (2.0)
**Change in beliefs about other students' orange juice liking**	−0.2 (0.9)	−3.6 (2.0)	−3.0 (2.4)
**Self-beliefs about**	7.3 (2.3)	6.0 (2.7)	7.6 (2.3)
**orange juice liking**			
**Self-beliefs about**	6.4 (2.6)	6.4 (3.1)	7.0 (2.7)
**apple juice liking**			

*Note: Standard deviations are presented in brackets*. Liking ratings: 0–10 cm line scale, anchors ‘don't like at all’ and ‘like very much’.

ANOVA also indicated an effect of condition on change to beliefs about other students' liking for orange juice [F (2, 81) = 25.3, p<0.05]. Participants in the negative in-group condition [p<0.05] and negative out-group condition [p<0.05] had a significantly greater reduction in rated beliefs about other students' liking for orange juice than the neutral group, suggesting that the manipulation was effective. No significant difference was observed between the negative in-group and negative out-group conditions [p = 0.24], suggesting that perceptions about others' liking of orange juice decreased to a similar extent in these groups. See Table 1.

### Self-beliefs about liking

ANOVA indicated a significant effect of condition on self-beliefs about liking of orange juice [F (2, 81) = 3.7, p<0.05]. As hypothesised, participants in the negative in-group condition had a lower rated liking of orange juice than the negative out-group condition [p<0.05] and the neutral in-group condition, although the latter did not survive a Bonferroni correction and was only marginally significant [p = 0.08].

No effect of condition was observed for evaluations of apple juice [F (2, 81) = 0.5, p = 0.60]. See Table 1. Because we observed that at baseline overweight and obese male students were perceived to like orange juice less than the two other conditions, we ran a further analysis with baseline perceptions as a covariate and found the exact same significant pattern of results.

## Study 1: Discussion

After exposure to negative social normative information suggesting other students dislike orange juice, participants tended to believe that they liked orange juice less than a group of participants who were exposed to neutral social information about orange juice. This effect was item specific in that information about liking of orange juice had no effect on liking for a similar drink (apple juice). The observed effect was also social group specific because self-beliefs about liking for orange juice were significantly lower when participants were provided with negative information about an in-group compared to an out-group. The results suggest that social normative information about a food/drink may change self-beliefs about liking.

## Study 2: Introduction

In Study 2, we wanted to examine whether social information would affect liking evaluations of a specific experience with a food. To ensure that the participants had a recent experience with the food to be evaluated, they consumed a small portion of the food at the start of the session. Study 2 also incorporated some minor modifications to improve on the design. First, the type of social information used differed from that used in Study 1 in that we used descriptions of another person's eating experience. We reasoned that exposing participants to a negative description of an actual experience with a food would be more similar to the type of negative social information individuals might normally encounter. In addition, we used a food rather than a drink to test for generalizability of the results. Finally, we included an additional measure to test whether changes in liking were associated with changes in perceptions about the food. At the end of the session, participants were served the exact same food sample again and we tested whether perceptions of similarity between the initial and later food samples differed according to condition.

## Study 2: Method

### Overview

Participants were led to believe that they would be taking part in a study involving reading about other students' experiences with food and making personality judgements. At the start of the session, the participants consumed and rated two foods (popcorn and a chocolate tea cake). They then read two other students' opinions about food and were told that these students had also consumed the chocolate tea cake. Depending upon condition, participants then read that the students thought the chocolate tea cake was average (neutral social information condition) or that the students did not like eating the chocolate tea cake (negative social information condition). After completing filler tasks, the participants were asked how much they themselves had liked eating the chocolate tea cake. The same foods were served again and participants rated how similar they were to the earlier samples.

### Participants

Forty-seven female psychology undergraduates from the University of Birmingham participated in exchange for course credit (19.8 years, SD = 3.0 years). To disguise the nature of the research, the study was advertised as ‘Consumer research on food preferences and personality’. Participants were instructed to abstain from eating for one hour prior to the study to ensure they were not satiated on arrival. Participants gave informed signed consent and the study protocol was approved by the University of Birmingham Research Ethics Committee. The study was conducted according to the ethical standards laid down in the Declaration of Helsinki 1964.

### Experimental Conditions

Participants were assigned to one of two conditions prior to arrival; negative social information (experimental) or neutral social information (control). In Study 1, we found that out-group information did not influence liking evaluations, therefore, we included only two conditions (negative and neutral social information) in Study 2.

In the negative social information condition, fictional accounts of the responses of two previous participants to questions indicated that they had not liked the chocolate tea cake (i.e. ‘*It wasn't very good, so overall I really didn't really enjoy it*’).

In the neutral social information condition, the responses of the previous participants were neutral concerning their experience with the chocolate tea cake, expressing no dislike (i.e. ‘*This was as enjoyable as I was expecting*’). The length of text was balanced across the two conditions. In neither condition did participants read about any student experiences with the non-target test food (popcorn). See supporting information S1 for full accounts.

### Test Foods

Participants consumed two foods at the start of the session: four pieces of toffee popcorn (Butterkist Toffee Popcorn, ×4 pieces = 12 g, 415 kcal per 100 g) and a Chocolate Tea Cake (Tunnock's Milk Chocolate Teacake Biscuits, ×1 = 25 g, 440 kcal per 100 g). These foods were also consumed at the end of the session when the participants made similarity judgements. These foods were chosen because a pilot study indicated that they were both were well-liked.

### Procedure

Participants were led to a testing room and completed questions on demographics and rated their baseline hunger; ‘how hungry are you right now?’ (mark with an x) using a 10 cm line scale with anchors ‘not at all’ and ‘extremely’. Participants were then informed that they would be required to eat and rate two food items. The researcher placed 4 pieces of toffee popcorn and a single chocolate tea cake on two separate plates on the table. Participants were instructed to first eat all of the popcorn and then make ratings; ‘the popcorn is sweet’ ‘the popcorn is enjoyable’ ‘the popcorn is dry’ on a 5 point likert scale; anchors ‘strongly disagree’ and ‘strongly agree’ and to follow the same procedure for the chocolate tea cake. The enjoyable rating made here is now referred to as ‘actual liking’.

After eating and rating the foods the researcher gave participants a booklet to read with instructions on the front *‘In this next section you are required to read information about the views of two students about food. It is important you read this information carefully, as you will be required to answer questions on these students later. You will be given 5 minutes to read the information as thoroughly as possible. Please turn over’*. The first half of the booklet was the same for all conditions; this included transcribed responses to 6 questions concerning eating habits for the two students (i.e. ‘what is your favourite food and why? *I really like Italian food, as it reminds me of family holidays and that part of the world.*’).

The second part of the booklet concerned student reports on their experience of eating the chocolate tea cake, although whether these were negative or neutral depended on the experimental condition. To corroborate the cover story, participants were then given another set of questionnaires to complete. The first 10 questions were judgements about the two fictional students, answered on 10 cm line scales; i.e. ‘this person is very introverted’ (anchors; disagree and agree). Included in these questions was a manipulation check that participants had noticed whether or not each student had disliked the food ‘*Student A would eat the tea cake again*?’ Next, participants completed questions concerning their own personality using the same scale (a total of 22 questions), i.e. ‘*I am very confident in new situations*’ and to corroborate the cover study, participants rated how often they ate a list of five snack foods (milk chocolate, dried fruit, biscuits, shortbread, toffee popcorn).

As we wanted to examine evaluated liking for the specific recent experience with the food, participants were then asked ‘think back to eating the food items earlier, how enjoyable were those food items?’ and rated the toffee popcorn and then the chocolate tea cake on a 10 cm line scale, anchors; ‘not at all’ and ‘extremely’. These ratings are referred to as later liking evaluations. The researcher then returned and gave participants a final questionnaire and the final samples of toffee popcorn and chocolate tea cake. Participants were instructed to sample each food and then rate how similar each sample was to the same food eaten at the start of the session, on 10 cm line scales, i.e. ‘*Compared to the first sample, this chocolate tea cake is*’, anchors; ‘not at all similar’ and ‘extremely similar’. Finally, participants completed the cognitive restraint scale of TFEQ [31], before being probed about the aims of the study, debriefed and having their weight and height measured (using digital scales and a stadiometer) to calculate BMI (kg/metres^2^).

### Analysis

To examine whether the two conditions differed in beliefs about how much the two fictional students had enjoyed the food, we collapsed the questions ‘Student A would eat the tea cake again’ and ‘Student B would eat the tea cake again’ for each participant and compared the groups using an independent sample t-test. To assess between-group differences for actual liking, later liking evaluations of the test foods and rated sample similarity were compared using independent sample t-tests.

## Study 2: Results

Five participants were close to guessing the aims of the study (i.e. *‘whether my liking of the food changed’*) and so were removed from analyses (the pattern of the results did not change). Mean baseline hunger/10 = 5.1, SD = 2.4. Mean restraint score = 7.0, SD = 4.7. Mean BMI was within the healthy range; 22.1, SD = 3.2.

### Manipulation check

The manipulation was successful because participants in the negative social information condition believed the other students did not like eating the chocolate tea cake as much as participants in the neutral social information condition: mean negative social information condition rating = 1.5, SD = 1.4; mean neutral social information condition rating = 6.6, SD = 1.2, [t(41) = 12.7, p<0.05].

### Actual liking and later liking evaluations

The two groups did not differ in their actual liking of either the toffee popcorn [t(41) = 0.5, p = 0.64] or chocolate tea cake [t(41) = 0.9, p = 0.34]. For the toffee popcorn, the food that had not been the subject of social information, later liking evaluations did not differ between the two conditions [t(41) = 0.5, p = 0.62]. For the target food, the chocolate tea cake, later liking evaluations did differ significantly between the two conditions. Participants in the negative social information condition believed they liked chocolate tea cake significantly less than participants in the neutral social information condition [t(41) = 2.1, p<0.05]. See Table 2.

**Table 2:** Study 2 Liking Ratings.

	Neutral social info condition, n = 21	Negative social info condition, n = 22
**Actual liking - Popcorn**	4.4 (0.7)	4.3 (0.8)
**Actual liking – Teacake (target food)**	4.1 (1.1)	3.8 (1.1)
**Later liking evaluation - Popcorn**	6.9 (2.2)	6.6 (2.1)
**Later liking evaluation - Teacake**	6.6 (2.4)	5.0 (2.6)

*Note: Standard deviations are presented in brackets*. Actual liking: 5 point likert, ‘strongly disagree’ to ‘strongly agree.’ Later liking: 0–10 cm line scale, anchors ‘not at all enjoyable’ and ‘extremely enjoyable’.

### Similarity ratings

Participants in the negative social information condition rated the second serving of chocolate tea cake as being significantly less similar to the first sample (6.6, SD = 2.9) than participants in the neutral social information condition (8.8, SD = 1.2) [t(41) = 3.3, p<0.05].There was no significant difference [t(41) = 1.8, p = 0.07] in rated similarity between the negative social information condition (7.9, SD = 2.2) and neutral social information condition (8.8, SD = 0.9) for the toffee popcorn.

## Study 2: Discussion

Participants who consumed a snack food and then read accounts of two other students who disliked eating that snack food, said they had liked the snack food less than participants who read neutral reports from other students about the snack. This effect occurred even though the two groups did not differ for their actual ‘online’ liking ratings of the snack. When later served the exact same type of snack food, participants exposed to the negative social information also believed it was less similar to the earlier serving they had consumed. One interpretation of this finding is that the mental representation of eating experience changed as a result of being exposed to others' negative evaluations of the food. Participants' evaluations about how they had liked eating another recently consumed snack food were not affected, suggesting that the observed effect was food item specific.

## General Discussion

Across two studies, we found that exposure to negative social information about a food or drink resulted in less positive liking evaluations than exposure to neutral social information about a food or drink. In Study 1, social normative information suggesting that an in-group do not like a drink tended to decrease beliefs about liking for that drink, but no effect was observed when the social normative information was about an out-group. In Study 2, reading others' negative accounts of disliking a snack food resulted in participants evaluating their recent experience with the same food less positively than a control group. Thus, social information can influence both beliefs about liking and evaluations of a recent eating experience.

The mechanisms by which social information influenced liking are not clear from the present studies. In Study 1, participants may have re-evaluated their beliefs about orange juice liking to adhere to the presented social norm and ‘fit in’ with the reference group. A shared identity with fellow students from the same university may have been a motivating factor, which is in keeping with other conformity and social norm research [28], [32]. Conversely, these results could be explained through Balance Theory, which posits that individuals have a desire to maintain consistency in their liking of other people and objects [33]. As individuals tend to like in-group members, learning that fellow in-group members were different to them and disliked orange juice may have created cognitive inconsistency (‘I like them and I like orange juice, but they don't like orange juice’). Thus, participants could have adjusted their liking evaluations in order to address this cognitive inconsistency. The finding that negative social normative information about an out-group did not reduce beliefs about liking would suggest that exposure to negative information about orange juice per se, may not be an explanation for the pattern of results. When interpreting our findings it is important to note that the manipulation check showed that both types of negative social information (in-group and out-group) caused equal changes to participant beliefs about others' liking of orange juice, so both groups did appear to be equally exposed to negative information about orange juice (albeit from different social groups). However, replicating our findings to show an increase in liking as a result of positive social information would test whether it is important that the information is social in nature.

Further work clarifying why negative social information about an in-group reduced liking more than negative social information about out-group is also warranted. This could be because participants did not identify with the out-group and/or because they disliked the out-group. Some research suggests that a number of negative stereotypes are associated with obesity, which would support the latter proposition [34]. For Study 1, the content of social information that brought about changes to liking beliefs also deserves further investigation. Participants were exposed to social normative information suggesting that other university students dislike drinking orange juice and rarely drink it. Although this is exactly the pattern of behaviour we would expect to see if a group did not like orange juice, it is not clear whether perceptions of others' consumption frequency and/or liking opinions influenced beliefs. It would be interesting to examine whether seeing other people avoiding a food (without explicit expression of dislike) would be sufficient to alter evaluated liking for that food.

In Study 2, we examined the effect of social influence on liking evaluations for a recent consumption experience. Participants in Study 2 were likely to be accessing their episodic memory for that recent experience to make liking judgements. Thus, a suggestion that could account for the findings in Study 2 is that the reports by the mock participants distorted event memory, which fits with the notion that memory can become distorted as a result of exposure to others providing inaccurate or false reports [35], [36]. By this account participants may have confused the two sources of information from memory (own vs. others' experience) and therefore had a memory that had become infused with both sources. As well as observing a significant reduction in evaluated liking after exposure to negative social information, participants also believed that a later identical sample of the test food was less similar to the earlier food sample they had consumed than participants in the control condition. We suggest that this provides some support for a memory distortion account, as the similarity judgements made between the two samples will have been reliant on accessing the earlier experience from memory. That being said, we did not measure memory content in the present study. An alternative explanation is that memory distortion did not occur and participants simply adjusted their liking evaluations to conform to the negative accounts. Exactly how or why this would also cause the decrease in similarity ratings is unclear though.

To date, there has been limited examination of how other people might influence evaluated liking of foods that have previously been eaten. As liking predicts food choice and intake [2] and many food decisions are made without direct sensory contact with food, liking evaluations are likely an important determinant of food choice [4]. Tentatively, we propose that knowing others dislike a food may reduce the likelihood of incorporating that food into our diet. Whether such findings could be applied to increase liking of food items by children and adults would be an interesting research avenue. The aim of the present studies was to examine if social influence can negatively alter food liking evaluations, but in the future it would be interesting to investigate whether liking evaluations for a food could be increased as a result of social influence. Findings elsewhere suggest that facial expressions of dislike produce stronger effects on desire to consume a food than expressions of enjoyment [15], so it may be the case that beliefs about liking follow a similar pattern.

### Limitations

In the present research we conducted two studies and tested different forms of social influence on liking evaluations in each study; social normative information about a social group's liking of a drink (Study 1) and others' accounts of an experience with a food (Study 2). We also examined the effect of social influence on two forms of liking evaluation separately in the two studies; liking beliefs (Study 1) and liking of a specific recent experience (Study 2). A cross over study using a single sample, to determine whether both kinds of social information influence both evaluation types would have been preferable, as this would allow us to better understand the relationships between these factors. We also only sampled university female students. As gender differences have previously been noted in social eating research [37], further work would be needed to examine if the present findings also apply to males. In future, the use of manipulation checks to examine group identity would also be advised. For example, we did not assess whether students in our study did strongly identify with other students (i.e. their in-group). However, there is evidence elsewhere that university membership promotes a strong sense of social identity and affiliation between students [38].

### Conclusions

Across two studies, we find that evaluations about food liking are prone to negative social influence. In Study 1, beliefs about liking of a drink were influenced by social normative information. In Study 2, exposure to others' negative accounts of eating a food decreased how much participants thought they had liked a recent consumption experience.

## Supporting Information

Supporting Information S1 **Mock participants' reports of food liking by condition for Study 2.**
(DOC)
