# Supplementary material for: Liking Food Less: The Impact of Social Influence on Food Liking Evaluations in Female Students
Source: PLoS One. 2012 Nov 14;7(11):e48858. doi: 10.1371/journal.pone.0048858 (PMC3498255; doi:10.1371/journal.pone.0048858)
Supplement: Supporting Information S1 — Mock participants' reports of food liking by condition for Study 2. (DOC) [file pone.0048858.s001.doc]

**S1**

*Neutral social information condition*

**Student A: Age = 19, Course = Undergraduate Psychology**

Were you looking forward to eating the Chocolate Tea Cake?

*Yes.*

Have you eaten Chocolate Tea Cake before?

*Yes*

How did the Chocolate Tea Cake taste?

*It was OK.*

How was the Chocolate Tea Cake compared to other times you have eaten it?

*This was as enjoyable as I was expecting.*

Would you eat the Chocolate Tea Cake again?

*I would probably eat it again.*

**Student B: Age = 20, Course = Undergraduate Psychology**

Were you looking forward to eating the Chocolate Tea Cake?

*I suppose.*

Have you eaten Chocolate Tea Cake before?

*Yes, a few times.*

How did the Chocolate Tea Cake taste?

*Quite good. Normally quite like it.*

How was the Chocolate Tea Cake compared to other times you have eaten it?

*It was similar to what I have eaten before.*

Would you eat the Chocolate Tea Cake again?

*Possibly.*

*Negative social information condition*

**Student A: Age = 19, Course = Undergraduate Psychology**

Were you looking forward to eating the Chocolate Tea Cake?

*Yes.*

Have you eaten Chocolate Tea Cake before?

*Yes*

How did the Chocolate Tea Cake taste?

*It wasn’t very good, it tasted cheap, so overall I really didn’t really enjoy it. Disappointing.*

How was the Chocolate Tea Cake compared to other times you have eaten it?

*This wasn’t as enjoyable or as nice as usual.*

Would you eat the Chocolate Tea Cake again?

*No.*

**Student B: Age = 20, Course = Undergraduate Psychology**

Were you looking forward to eating the Chocolate Tea Cake?

*I suppose.*

Have you eaten Chocolate Tea Cake before?

*Yes, a few times.*

How did the Chocolate Tea Cake taste?

*Not enjoyable. Normally quite like it, but can’t say this was good at all.*

How was the Chocolate Tea Cake compared to other times you have eaten it?

*It wasn’t as nice a tea cake to what I have eaten before.*

Would you eat the Chocolate Tea Cake again?

*I would avoid eating it most probably.*
